# A Rare Case of Haemophilus Influenzae Serotype F Endocarditis Complicated by Concurrent Cardiogenic and Septic Shock: A Case of Challenging Management

**DOI:** 10.3390/jcdd9110384

**Published:** 2022-11-09

**Authors:** Michael Kwok, Wasiq Sheikh, Fabio V. Lima, Raymond Russell

**Affiliations:** 1 Divison of Biology and Medicine, Warren Alpert Medical School of Brown University, 222 Richmond Street, Providence, RI 02903, USA; 2 Cardiovascular Institute, Warren Alpert Medical School of Brown University, Providence, RI 02903, USA

**Keywords:** septic shock, cardiogenic shock, acute decompensated heart failure, valvular disease, endocarditis

## Abstract

*H. Influenza* is a rare cause of endocarditis. We report a case of *H. Influenza* endocarditis that was complicated by cardiogenic and septic shock, active myocardial ischemia and aortic insufficiency. The goal of this report is to help recognize the signs and symptoms of endocarditis and to discuss management strategies in patients with concomitant cardiogenic and septic shock complicated by aortic insufficiency.

## 1. History of Presentation

A 69-year-old male presented to the hospital with a fever and chills one week after being discharged for treatment of *Haemophilus influenza* bacteremia, completing a two-week course of cefuroxime. During his prior admission, he underwent a transthoracic echocardiogram (TTE) to determine his heart’s ejection fraction (EF) as he was starting to appear volume overloaded. The TTE showed EF of 55%, mild aortic stenosis, and moderate aortic insufficiency, which was not further investigated despite positive blood cultures for *Haemophilus influenza*. Other prior workup included a normal chest X-ray, a CT scan showing minimal tree-in-bud opacities, and an MRI of the ankle revealing signs of synovitis.

During this presentation, he arrived to the emergency department with “achy”, non-radiating, non-reproducible chest pain. His vitals revealed a temperature of 101 °F, a heart rate of 127 bpm, blood pressure of 67/39 mm Hg, and an oxygen saturation of 96% on room air. A physical exam on admission was notable for a grade 3 systolic ejection murmur heard best at the right upper sternal border without radiation to the carotids. The peripheral exam was free of any Janeway lesions or Osler nodes.

## 2. Past Medical History

Coronary artery disease status-post posterior descending artery stent in 2007 (unknown if associated with myocardial infarction), 50 packets of cigarettes per year smoking history, hypertension, hyperlipidemia, chronic lower back pain, and emphysema. 

## 3. Differential Diagnosis

Shock (septic shock +/− cardiogenic shock), endocarditis, cardiomyopathy, pneumonia.

## 4. Investigations

**Figure 1:**
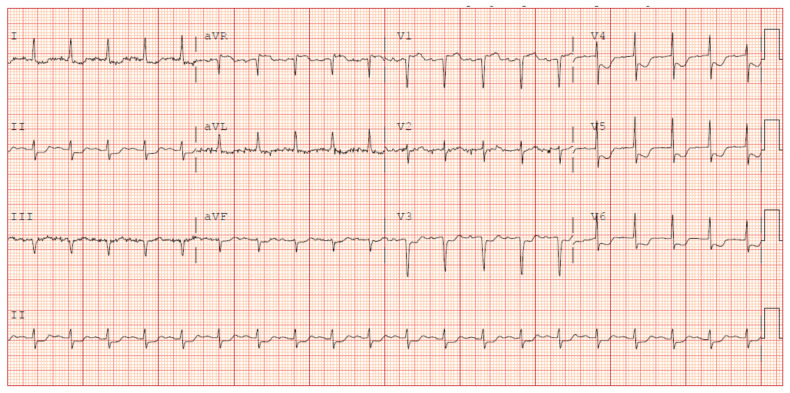
Initial EKG on presentation to the ED demonstrating diffuse ST segment depressions.

**Figure 2:**
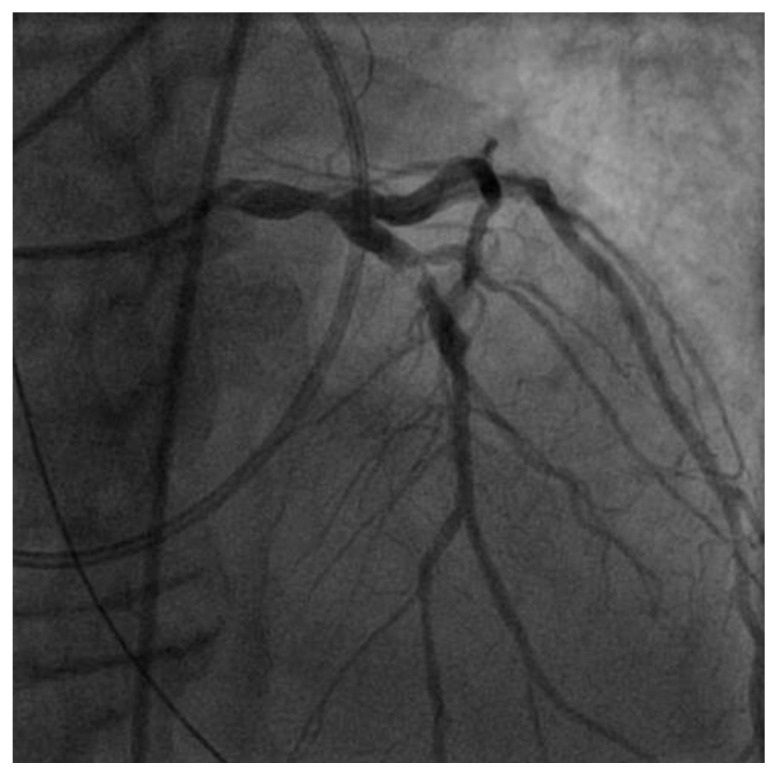
Right anterior oblique coronary angiography demonstrating 80% left main coronary artery and 90% proximal left anterior descending coronary artery stenosis.

**Figure 3:**
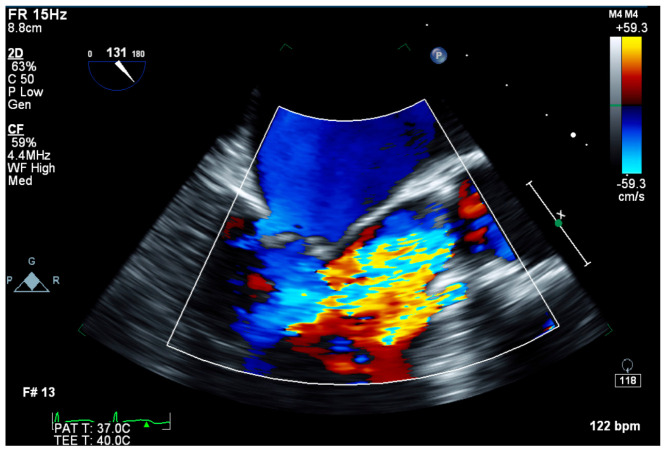
Transesophageal echocardiogram long axis view with color doppler demonstrating two turbulent jets, consistent with severe aortic insufficiency.

**Figure 4:**
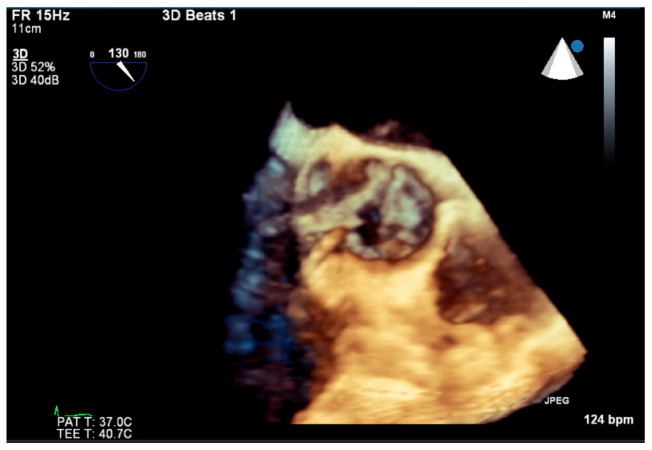
Three-dimensional reconstruction of transesophageal echocardiogram mid-esophageal aortic valve short-axis view demonstrating aortic valve destruction with a large hole in the center.

**Figure 5:**
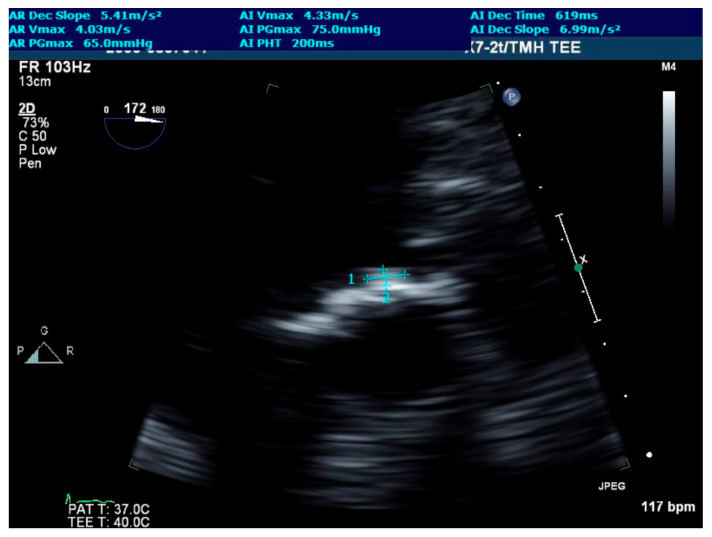
Zoomed-in transesophageal echocardiogram deep transgastric view of the aortic valve from the left ventricular outflow tract. Cross-hairs depict the mass on the aortic valve.

Initial laboratories revealed leukocytosis and an elevated troponin to 20.6 ng/mL. The initial electrocardiogram was notable for diffuse ST-depressions (Figure 1). The chest X-ray showed bibasilar patchy airspace disease. Bedside TTE showed anterior wall hypokinesis and an ejection fraction of 25–30%. Blood cultures drawn on admission were again positive for beta-lactamase negative H. influenzae serotype F. The patient underwent a left heart catheterization which showed a severe distal left main lesion into the proximal left anterior descending artery and circumflex but was noted to have TIMI 3 flow throughout (Figure 2). Right heart catheterization demonstrated a cardiac index (CI) of 2.1 L/min/m^2^, pulmonary capillary wedge pressure (PCWP) of 23 mmHg, right atrial pressure of 15 mmHg, and a systemic vascular resistance of 900 dynes. The patient then underwent a transesophageal echocardiogram (TEE) which revealed severe aortic insufficiency (Figure 3), valvular destruction (Figure 4), and notably, an aortic valve mass consistent with endocarditis (Figure 5).

## 5. Management (Medical/Interventions)

In the MICU, the patient was treated for concomitant cardiogenic and septic shock given his low PVR, high PCWP, and low CI. On the first day, vasopressors, inotropic support and broad-spectrum antibiotics were initiated. The patient also developed acute pulmonary edema requiring intubation and aggressive diuresis. Given evidence of active ischemia secondary to severe multivessel atherosclerosis as evidenced by the left heart catheterization, unfractionated heparin was initiated. The following day, the patient’s PCWP and vital signs improved resulting in extubation and discontinuation of the vasopressors and inotropic support. Due to his low CO in the setting of cardiogenic and septic shock, mechanical support by an intra-aortic balloon pump (IABP) and catheter-based ventricular assist devices were considered but eventually not implemented due to contraindications with severe aortic insufficiency and endocarditis. Extracorporeal membrane oxygenation (ECMO) was not initiated as the patient started to demonstrate improving clinical stability, was extubated, and weaned off the vasopressors and inotropic support. The subsequent LVEF was 40% by TTE. Due to elements of hemodynamic stabilization at the time, surgical intervention was initially deferred until post-bacterial clearance with antibiotics. Unfortunately, after 2 weeks the patient developed acute hypotension and pulmonary edema requiring re-initiation of vasopressors and inotropic support, and emergent surgery with aortic valve replacement and two-vessel coronary artery bypass grafting. Post-operatively, he recovered at an appropriate pace, was discharged, and transitioned to outpatient cardiac rehabilitation with a six-week course of ceftriaxone.

## 6. Discussion—Association with Current Guidelines/Position Papers/Current Practice

This case highlights two important learning points: first, the consideration of atypical endocarditis is important especially in patients with known compromised valves and a recent history of bacteremia with an atypical organism. Second, this case illustrates the clinical difficulties in treating both septic and cardiogenic shock in the setting of endocarditis and aortic insufficiency.

The HACEK classification of bacteria was designed to encompass *Haemophilus* species. However, given that *H. Influenzae* is rarely cultured in cases of infective endocarditis, the “H” is now more commonly used for *Haemophilus* species *other* than *H. Influenzae* [1,2]. Non-typeable *H. Influenzae* infections make up a majority of infections and are associated with higher mortality rates in adults, but recent epidemiological surveys note that serotype F infections are increasing in patients >60 years [3,4,5]. There is, however, a paucity in the literature regarding endocarditis caused by *H. influenzae* of any serotype and cases are limited to case reports or vignettes [6,7]. Our case demonstrates the need to maintain endocarditis on the differential diagnosis when a patient with a recent history of bacteremia presents as being acutely ill again despite completing antibiotic therapy for prior infection. Prior knowledge of this patient’s valvular disease on the TTE in the setting of septic and cardiogenic shock prompted further investigation with the TEE to better assess valvular function. This led to visualization of the aortic valve vegetation and severe aortic insufficiency.

A hallmark of septic shock is the imbalance of oxygen supply and a demand which inevitably places a strain on the myocardium leading to both cardiac and global tissue hypoxia. In our patient’s case, his advanced aortic valve disease and triple-vessel atherosclerosis limited his heart’s ability to match the increased oxygen demand and resulted in concurrent cardiogenic shock. We were able to achieve hemodynamic stability with vasopressors and inotropes, however, alternative modalities including catheter-based assist devices (such as Impella) and IABP were considered. However, knowledge of the aortic valve vegetation and degree of insufficiency deemed it too high of a risk for septic emboli and worsening of the aortic regurgitation with Impella or IABP, thus mechanical support was not initiated. ECMO was also considered and has been described in a few case reports of mechanical dysfunction and hemodynamic compromise [8]. However, in the setting of left ventricular dysfunction and a high degree of coronary atherosclerosis, ECMO can worsen cardiac dysfunction due to retrograde non-pulsatile blood flow and coronary hypoxia from lower oxyhemoglobin saturation in the upper body with use of a femoral arterial line in a veno-arterial ECMO system [9,10,11].

## 7. Follow-Up

The patient was seen in clinic nearly one month after discharge and was recovering well. Pathology analyses of the aortic valve demonstrated evidence consistent with endocarditis inclusive of prominent nodular calcification, focal inflammation, necrosis, and microabscess formation.

## 8. Conclusions

*H. Influenzae* serotype F confirmed by bacteriological testing is a very rare cause of endocarditis. This diagnosis should be suspected in patients with valvular dysfunction, prior episodes of *H. Flu* serotype F bacteremia, and a new onset fever. Early TEE should be utilized to assess for valvular function and complications in these patients. Mechanical support can be considered in patients with mixed shock; however, limitations remain among patients with severe aortic valve insufficiency and endocarditis. ECMO is an alternative support measure that may be employed, however, in patients with high degrees of ventricular dysfunction and atherosclerosis, close monitoring for decompensation of cardiac function is necessary. Medical therapies and close intensive care monitoring are the focus of care for such patients. If the patient continues to decompensate, immediate surgical replacement of the infected valve should be considered.

## Data Availability

Not applicable.
